# Individualized treatment for acute Achilles tendon rupture based on the Copenhagen Achilles Rupture Treatment Algorithm (CARTA): a study protocol for a multicenter randomized controlled trial

**DOI:** 10.1186/s13063-020-04332-z

**Published:** 2020-05-12

**Authors:** Maria Swennergren Hansen, Marianne Toft Vestermark, Per Hölmich, Morten Tange Kristensen, Kristoffer Weisskirchner Barfod

**Affiliations:** 1 Physical Medicine and Rehabilitation Research – Copenhagen (PMR-C), Department of Physiotherapy, Copenhagen University Hospital, Kettegård Alle 30, 2650 Amager-Hvidovre, Denmark; 2 Sports Orthopedic Research Center – Copenhagen (SORC-C), Department of Orthopedic Surgery, Copenhagen University Hospital, Kettegård Alle 30, 2650 Amager-Hvidovre, Denmark; 3 Department of Orthopedic Surgery, Viborg Regional Hospital, Heiberbs Allé 4, 8800 Viborg, Denmark; 4 Department of Orthopedic Surgery, Copenhagen University Hospital, Amager-Hvidovre, Denmark

## Abstract

**Background:**

An individualized treatment algorithm (Copenhagen Achilles Rupture Treatment Algorithm (CARTA)) based on the ultrasonographic appearance of an acute Achilles tendon rupture has been developed aiming to select the correct patients for operative and non-operative treatment. The objective of this study is to investigate if this individualized treatment algorithm gives a better functional outcome than treating all patients either operatively or non-operatively per default.

**Methods/design:**

This study is conducted as a multicenter, three-armed randomized controlled trial. Participants are included from four hospitals in Denmark and randomized 1:1:1 to one of three parallel groups: 1) Intervention group—participants are treated according to an individualized treatment algorithm; 2) Control group A—participants are treated non-operatively; 3) Control group B—participants are treated operatively. The individualized treatment algorithm for the intervention group is based on an ultrasonographic examination; tendon overlap and elongation below 7% is to be treated non-operatively, while no tendon overlap and/or elongation above 7% will be treated operatively. Over a period of 3 years, 300 participants will be included. The primary outcome is the heel-rise work test at 12 months post-injury. Secondary outcomes are tendon elongation, the Achilles tendon Total Rupture Score (ATRS), the rate of re-ruptures, and other complications. The primary analysis will be conducted as an intention-to-treat analysis.

**Discussion:**

This trial will indicate if treatment of acute Achilles tendon rupture can be individualized based on elongation and tendon overlap. It is hypothesized that different patients will benefit from different treatments instead of offering all the same treatment.

**Trial registration:**

ClinicalTrials.gov, NCT03525964. Registered 16 May 2018.

## Background

Acute Achilles tendon rupture is an increasingly common injury during both sports and recreational activities [1]. Numerous studies investigating operative versus non-operative treatment with a focus on re-rupture rate and complications related to the treatment have been performed. Overall, non-operative treatment results in a higher re-rupture rate, which is argued to be acceptable due to the lower rate of serious complications such as sural nerve damage and deep infections [2–6]. As a consequence, recent studies have focused on refining non-operative treatment of acute Achilles tendon ruptures [7–9]. Simultaneously, standard treatment of acute Achilles tendon rupture has shifted from operative to non-operative in many Scandinavian departments [1, 10], though the impact on functional outcome of this change toward non-operative treatment is not yet clear [11, 12].

It is likely that some patients with primary Achilles tendon rupture need surgery and some are better off with non-operative treatment. This could be due to the individual morphology of the ruptured tendon and the potential to heal with good strength and a functionally acceptable length [13]. The identification and selection of patients for operative treatment has not yet been well investigated [14, 15]. The clinical criteria for recommending operative treatment differ and have not been derived from evidence. Some clinicians would recommend operative treatment for young patients with high functional demands and others would say that this should be avoided if the patient has a poor prognosis of healing based on comorbidities. These aspects clearly need to be taken into consideration but cannot stand alone. A quantitative and evidence-based measure or method is needed to guide the clinician for optimal treatment. Amlang et al. [14] have developed an ultrasonographic classification of Achilles tendon rupture but have not evaluated the length of the tendon or functional outcome after healing, and the method demands an experienced examiner. Barfod et al. have developed and validated the ultrasound-based Copenhagen Achilles Length Measure (CALM), which can be conducted in the emergency department as part of the primary diagnosis [16, 17]. Barfod et al. have also investigated the correlation of Amlang’s classification and CALM in the acute setting to functional outcome scores after 1 year in non-operative-treated acute Achilles tendon rupture. CALM was able to predict the final length of the tendon at one-year follow-up (manuscript in press). CALM is the base of the newly developed Copenhagen Achilles Rupture Treatment Algorithm (CARTA) together with examination of tendon overlap inspired by Amlang’s classification system [14], which may be used as a predictive tool for identifying patients at risk of critical elongation of the tendon after healing and poor functional outcome. A manuscript on CARTA has been submitted to a scientific journal.

Based on CARTA we propose an evidence-based, individualized algorithm for treatment of acute Achilles tendon rupture. The objective of this trial is to investigate if this algorithm is superior to both operative and non-operative treatment.

Hypothesis: Patients with acute Achilles tendon rupture treated in accordance with the individualized treatment algorithm (CARTA) will have a better limb symmetry index for heel-rise-work compared to patients treated either non-operatively or operatively per default.

Null hypothesis: There is no difference in limb symmetry index for heel-rise-work between patients treated in accordance with the individualized treatment algorithm (CARTA) and patients treated non-operatively or operatively, respectively.

## Methods/design

This is a report of the second version of the trial protocol dated 12 December 2018. Any important protocol modifications will be addressed at ClinicalTrials.gov (NCT03525964), to the Ethical Review board, and in the *Trials* journal. The protocol was developed in accordance with the guidelines and checklists for Standard Protocol Items: Recommendations for Interventional Trials (SPIRIT; Additional file 1) and Consolidated Standards of Reporting Trials (CONSORT).

### Design

The trial is performed as a randomized controlled trial with patients included from four hospitals in Denmark (Copenhagen University Hospital Amager-Hvidovre, Hospital Little Belt Kolding, Viborg Regional Hospital, and Zealand University Hospital, Køge). The participants are individually randomized in a 1:1:1 order in blocks stratified by hospital to one of three parallel groups:

1. Intervention group: Participants are treated according to an individualized treatment algorithm
2. Control group A: Participants are treated non-operatively
3. Control group B: Participants are treated operatively

### Objective

The primary objective of the trial is to investigate if individualized treatment of acute Achilles tendon rupture (CARTA) is more effective than treating all patients either operatively or non-operatively per default. The CARTA allocates participants to either operative or non-operative treatment based on the ultrasonographic measurement CALM.

### Primary outcome measure

The primary outcome is the heel-rise work test, which is an endurance test where the participant stands on one leg and lifts the heel up and down until exhaustion [18]. The number of heel rises is counted and the height measured and plotted into a diagram on the x-axis and y-axis, respectively. Based on the weight of the participant the total work is estimated as area under the curve. The heel lift distance between the heel and the floor is measured in millimeters. The procedure is performed on the uninjured leg first and subsequently on the injured leg at 12 months. The participant is barefoot for the heel-rise work test and stands on a flat surface with a 10° inclination. The measurement system MuscleLab (Ergotest Technology, Oslo, Norway) is used at all the centers for these functional tests. The tests are developed by the Gothenburg group [18]. The metric for both the primary outcome measure as well as all the secondary outcomes is value at time point, meaning that it is the difference at a point in time between groups that will be assessed. The method of aggregation (how data from each group will be summarized) will be equal for all three groups and will be either mean, median, percent, or proportion depending on the results.

### Secondary outcome measures

**Fig. 1:**
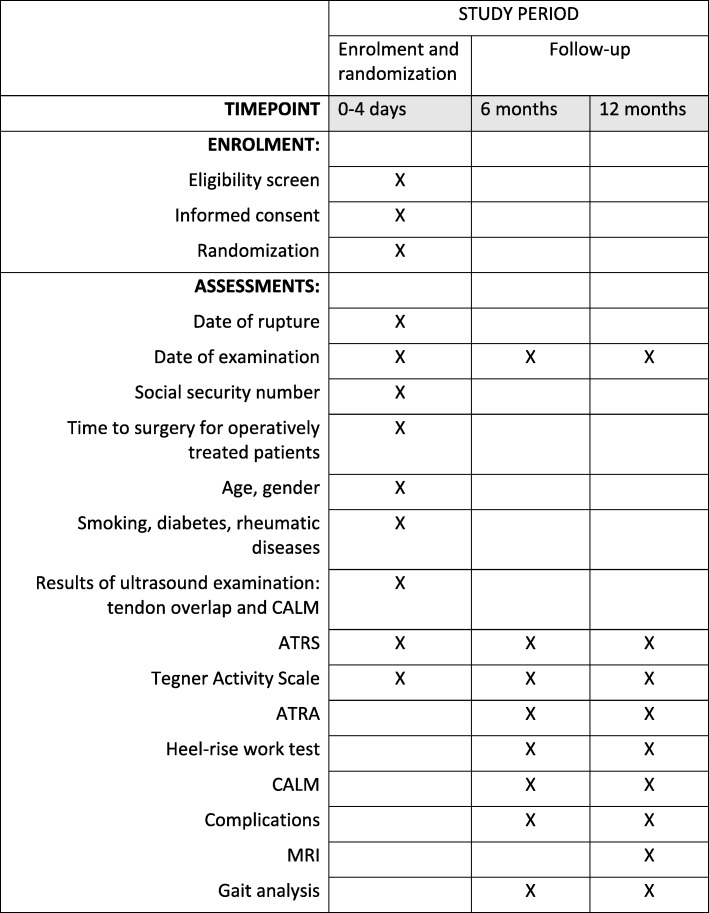
SPIRIT table of enrolment and assessments. *CALM* Copenhagen Achilles Length Measure, *ATRS* Achilles tendon Total Rupture Score, *ATRA* Achilles Tendon Resting Angle, *MRI* magnetic resonance imaging

The secondary outcome measures are listed below (the timing of the assessments is shown in Fig. 1).

**Fig. 2:**
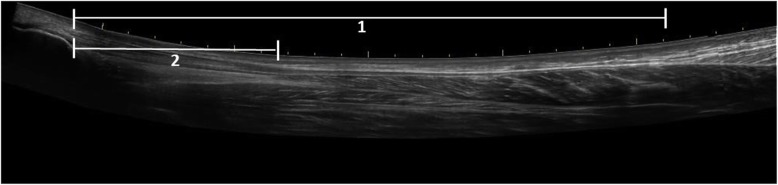
Copenhagen Achilles Length Measure (CALM): an ultrasonographic measurement of tendon elongation. CALM comprises two measurements: the total length of the Achilles tendon (distance 1) and the free length of the Achilles tendon (distance 2)

- Copenhagen Achilles Length Measure (CALM) [19] is an ultrasonographic measurement of tendon elongation. It comprises two measurements (Fig. 2): the total length of the Achilles tendon (free tendon and fascia) and the free length of the Achilles tendon. The measurements are preformed the same way; they have the same distal landmark but the proximal landmark differs. CALM has shown good reliability and is recommended over other measurements of elongation [17, 20, 21]. The measure is performed as described by Barfod et al. but with the feet of the patient (laying in the prone position) hanging free of the table instead of with 10° degrees of plantar flexion in the ankle joints as originally described [16, 21]. Landmarks are identified and marked on the skin. The distal landmark is the posterior and most superior part of the calcaneus in the midline, which on sagittal ultrasound examination is identified as the point where the cortical bone and its underlying shadow end. The proximal landmark for the total length of the Achilles tendon is the distal tip of the medial gastrocnemius head, which on sagittal ultrasound examination is recognized as the point where the most distal muscular fibers are inserted into the deep crural fascia. The proximal landmark for the free length of the Achilles tendon is the distal tip of the soleus muscle, which is defined at the point where the most distal muscular fibers insert into the Achilles tendon. The distance between these two points on the un-injured leg defines the total length of the Achilles tendon and the free length of the Achilles tendon, respectively. The differences between the non-injured and the injured leg are defined as tendon elongation. At inclusion only the total Achilles tendon length is measured for both legs. The examination of both the total and the free part of the tendon of both the injured and un-injured leg is performed at 6- and 12-month follow-up. The position of the feet also differs between inclusion and follow-up. During inclusion, the originally described position of the feet is used (10° of plantar flexion in the ankle joints) but during the 6- and 12-month follow-up, the feet hang free of the table.
- Heel-rise work test: The test is conducted as described for the primary endpoint and conducted as a secondary endpoint at 6 months.
- Achilles tendon resting angle (ATRA) [22]: ATRA is an indirect measurement of tendon elongation. The patient lies flat in the prone position on the examination bed. The knee is flexed at 90 degrees and the ankle sits in a relaxed position. The ATRA is determined as the angle between the longitudinal axis of corpus of the fibula and the longitudinal axis of corpus of the fifth metatarsus. ATRA is determined for both the injured and the uninjured leg. The difference between the healthy and the injured leg is evaluated. ATRA has shown excellent reliability [22, 23].
- Complications: Incidence rates of the commonly known complications and adverse events such as infection, re-rupture, permanent sural nerve dysfunction, deep venous thrombosis, adhesions, and pain at the scar are noted. Attention is also paid to unknown complications in relation to the treatments.
- Achilles tendon Total Rupture Score (ATRS) [24]: Patients fill out the ATRS questionnaire at inclusion and after 6 and 12 months in connection with the follow-up visits in the outpatient clinic. ATRS is a patient-reported outcome measure developed to assess symptoms and physical activity after treatment for acute Achilles tendon rupture. The Danish version was found to have good validity and be reliable to use for comparison on the group level [25].
- Tegner Activity Scale: A scale that aims to provide a standardized method of grading work and sporting activities. Tegner activity scale is a graduated list of activities of daily living, recreation, and competitive sports. The patient is asked to select the level of participation that best describes their level of activity at inclusion and at 6 and 12 months after rupture and before injury [26].
- Calf circumference: The patient sits on an examination bed with legs hanging down. The circumference of the calf is measured using a measuring tape (13 cm under apex patella).
- MRI: MRI of the calf is performed after 1 year for the first 60 patients included at Copenhagen University Hospital Amager-Hvidovre. MRI will be conducted to obtain a deeper understanding of changes to the different structures in the muscle–tendon complex during the first year after rupture. The MRI will be conducted at Copenhagen University Hospital Amager-Hvidovre in an Acanto 1.5 T scanner. Both lower legs will be scanned.
- Gait analysis: Gait analysis is performed after 6 and 12 months for the first 60 patients included at Copenhagen University Hospital Amager-Hvidovre. The gait analysis will be conducted at the Gait Laboratory, Copenhagen University Hospital Amager-Hvidovre. Twenty-two reflective markers are placed on the patient’s skin with tape on specific anatomical locations, as described by Speedtsberg et al. [27]. The participants are instructed to walk barefoot at a self-selected speed on a 10-m level walkway. The reflective markers are filmed by eight infrared cameras, whereby the joint angles during gait are measured. In combination with the ground reaction forces from two force plates embedded in the floor, the joint moments and powers will be calculated.

### Study participants

This is a multicenter trial. The participants are patients who are treated for acute Achilles tendon rupture at Copenhagen University Hospital Amager-Hvidovre, Viborg Regional Hospital, Hospital Little Belt Kolding, and Zealand University Hospital Køge. Patients who do not wish to participate are treated non-operatively.

#### Inclusion criteria

- 18 to 65 years of age
- Appointment in the outpatients department within 4 days of injury
- Total Achilles tendon rupture
- Initial treatment with split plaster cast with the ankle in maximal plantar flexion must be started within 24 h of injury
- The patient must be expected to be able to attend rehabilitation and post-examinations
- The patient must be able to speak and understand Danish
- The patient must be able to give informed consent

#### Exclusion criteria

- Rupture of the Achilles tendon either at the insertion on the calcaneus or at the musculotendinous junction of the triceps surae
- Previous rupture of the Achilles tendon in any of the two legs
- Treated with flourquinoles or corticosteroids within the last 6 months
- Being medically treated for diabetes
- Other conditions prior to the injury resulting in reduced function of any of the two legs
- Contraindication for surgery: severe arthrosclerosis with no palpable pulse in the foot, broken skin in the Achilles region of the injured leg
- Inability to lie in the prone position on the operating table
- Terminal illness or severe medical illness: American Society of Anesthesiologists (ASA) score ≥ 3

### Recruitment organization

The participants attend the outpatient clinic within 4 days post-injury. They are examined by an investigator to assess if they can be included. If possible, the participants are informed of the study, verbally and in writing. The participants are given the opportunity to decide whether they want to participate in the study and are informed of their right to consider this for 24 h.

### Randomization

Randomization is computer based following the random allocation rule to ensure balanced group sizes. Randomization is facilitated through a web-based database hosted by Procordo, Copenhagen, Denmark. The software will produce an allocation number for the patient to one of the groups: 1) intervention group, 2) control group A, or 3) control group B. Block randomization will be conducted with the participants individually randomized in a 1:1:1 order in blocks stratified by hospital to one of three parallel groups. The allocation key is stored by Procordo and only accessible by the project contact person at Procordo.

### Blinding

The investigators performing the functional follow-up evaluation are partly blinded to the intervention. Patients will wear tape over the Achilles region on the injured leg during the functional tests. The data from the blinded part of the examination are secured before the ultrasound examination is performed. The ultrasound examination is performed semi-blinded as it is impossible to cover the surgical scar during the scan. This examination is performed last. The investigator knows if the patient is treated operatively or not but does not know to which group the patient has been allocated. The data analysis will be conducted fully blinded. The patients are unblinded throughout the trial.

### Setup

#### Places of investigation

The study is taking place at Copenhagen University Hospital Amager-Hvidovre, Viborg Regional hospital, Hospital Little Belt Kolding, and Zealand University Hospital Køge. The standard treatment at these hospitals is the same. In general, patients are offered non-operative treatment with the following relative indications for operative treatment: (1) degenerative ruptures (patients treated with steroids or fluorquinolones within the past 6 months); (2) delayed diagnosis and start of treatment (equinus cast) of more than 24 h; (3) re-rupture; (4) avulsion-type rupture; (5) strong wish for operative treatment from the patient.

### Treatment

An overview of how the treatment will proceed during the first year is described in Table 1.

**Table 1:** Treatment overview. *CARTA* Copenhagen Achilles Rupture Treatment Algorithm, *CALM* Copenhagen Achilles Length Measure, *ATRA* Achilles Tendon Resting Angle

Visit	Time point	Place	Scheduled intervention
**1**	Day 0	Emergency Department *Surgeon*	Diagnosis of Achilles tendon rupture. The injured leg will be placed in a split plaster cast with the ankle in maximal plantar flexion achieved by placing the patient in prone position on the examination bed.
**2**	0-4 days	Outpatient clinic *Surgeon or physiotherapist*	Inclusion, informed consent, randomization and decision of treatment based on CARTA.
			1) For the patients allocated individualized treatment the finding of ultrasonographic evaluation decides further treatment of non-operative and operative regime.
			2) Patients allocated non-operative treatment will be treated with a circular below-the-knee cast
			3) Patients allocated operative treatment will be scheduled and prepared for surgery.
**3**	0-14 days	Operating theatre *Surgeon*	Operation of the patients appointed for operative treatment performed or supervised by trained orthopedic surgeon specialized in Foot- and Ankle, Traumatology or Sports Traumatology.
**Counting of weeks starts at “day of randomization” if non-operative treatment or at “day of surgery” if operative treatment.**			
**4**	3 weeks	Outpatient clinic *Nurse* Physiotherapist	Removal of cast and any sutures. Injured leg placed in functional brace with 20 degrees plantar flexion of the ankle. Instructions of limited, protected movements over the ankle and successive removal of two wedges at 2 weeks interval.
**5**	9 weeks	Outpatient clinic Physiotherapist	Functional brace is removed, and the tendon is examined. Instructions by physiotherapist of rehabilitation from 9 weeks and onwards.
**6**	6 months	Outpatient clinic *Physiotherapist*	Follow-up evaluation with Heel-Rise Work Test, CALM and ATRA.
**7**	12 months	Outpatient clinic *Physiotherapist*	Follow-up evaluation with Heel-Rise Work Test, CALM and ATRA.

#### Diagnosis

A patient history of a snap or strike at the back of the calf followed by difficulty in walking. Clinical examination reveals a defect in the Achilles tendon 3–6 cm proximal to the calcaneus. The calf-squeeze test [28] reveals no plantarflexion and the Matles test [29] reveals increased dorsiflexion of the ankle in the injured limb. Finally, the patient is unable to perform a single heel rise on the injured side.

#### Initial treatment until appointment in the outpatient department

When clinical diagnosis is made in the emergency department, a split plaster cast with the ankle in maximum plantar flexion is applied. No weight bearing is allowed.

#### Anti-thrombotic treatment

The risk of a thromboembolic event is increased for patients with acute Achilles tendon rupture [30]. Patients with one or more predisposing factors for a thromboembolic event will be offered anti-thrombotic treatment of 10 mg rivaroxaban orally for the initial 21 days of treatment.

Known predisposing factors:

- Previous thromboembolic event (deep venous thrombosis/pulmonary embolism)
- Thrombophilia (deficit of anti-thrombin, protein S or C, homozygous factor V Leiden, and Lupus anti-coagulopathy)
- Previous or present cancer
- BMI > 40
- First-degree relative with previous pulmonary embolism

#### The Copenhagen Achilles Rupture Treatment Algorithm (CARTA)

**Fig. 3:**
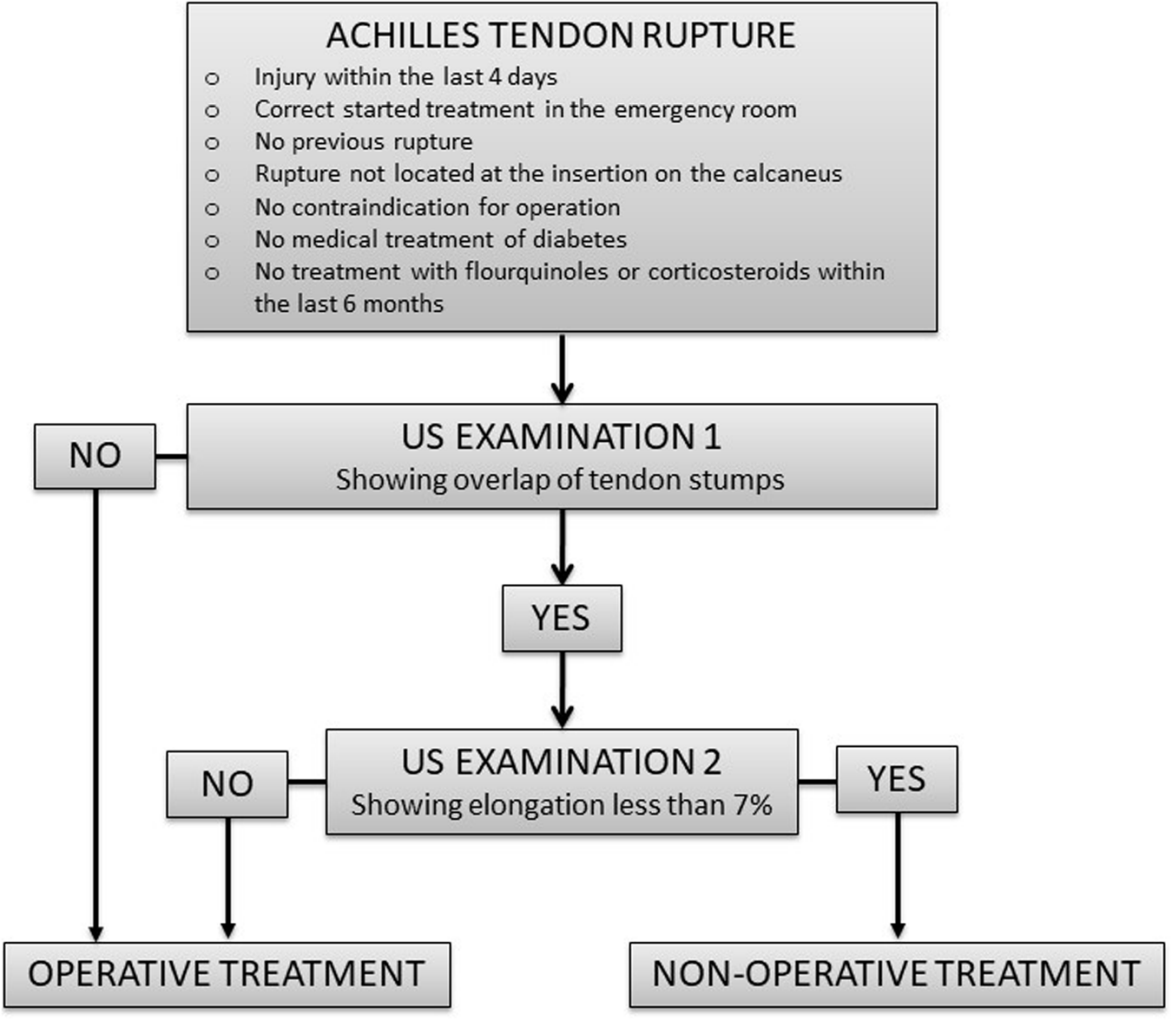
The Copenhagen Achilles Rupture Treatment Algorithm (CARTA) based on two ultrasonographic (US) examinations

Participants randomized to the intervention group will be treated according to CARTA (Fig. 3). A paper describing development of CARTA has been submitted for publication. The ruptured tendon is ultrasonographically examined and this is divided into two steps. Firstly, the degree of overlap at the site of rupture is investigated inspired by Amlang’s Classification system [14] and, secondly, the elongation of the tendon is estimated by CALM. The patient lays prone on the examination table with 10–20° flexion of the knee and 10° plantar flexion over the ankle or matching angulation to the contralateral ankle. The rupture is located in a longitudinal scan. The location where the tendon appears to be ruptured is found and the probe is rotated 90 degrees to a transverse picture. The cross-sectional area is investigated. The probe is then moved proximally–distally as well as in the caudal–cranial direction. If the examiner can identify a transverse picture with less than 25% fibers of the cross-sectional area, the rupture is evaluated as not overlapping. If more than 25% fibers of the cross-sectional area, the tendon is evaluated as overlapping. Tendon overlap is the first part of the algorithm that must be fulfilled to accept non-operative treatment, otherwise an operation is indicated.

Secondly, the elongation is measured by the CALM measure. Both legs are examined and the difference between the sides calculated as the elongation. The elongation is given in percentage of the length of the non-injured tendon. Patients with up to 7% elongation of the tendon are treated non-operatively and patients with 7% elongation or more are treated operatively. An elongation of 7% or more at time of injury can predict elongation of 10% or more at 1 year with a sensitivity of 77% and a specificity of 50% (paper submitted for publication). CALM is performed as previously described under outcome measures, but when used within the algorithm, the patient is positioned as described for the first part of the ultrasound examination.

#### Non-operative treatment

The participants destined for non-operative treatment, either through allocation to the non-operative group or as a result of allocation to the individualized treatment algorithm, are treated with a circular below-the-knee cast from the time of the first appointment in the outpatient clinic. The ankle is held at maximal, unforced plantar flexion. Weight bearing is not allowed and crutches are obligatory. After 3 weeks from initiated treatment in the emergency department the cast is removed in the outpatient clinic and the injured leg is transferred to a functional brace (Walker boot) with three heel wedges promoting 20° plantar flexion over the ankle. The wedges are inspired by the Breg Achilles wedges and reach from the heel to the metatarsal bones. They come in two sizes, medium (length 15 cm and height 2 cm each) and large (length 19 cm and height 2.5 cm each).

The patient will follow standard rehabilitation and the follow-up evaluations.

#### Operative treatment

**Fig. 4:**
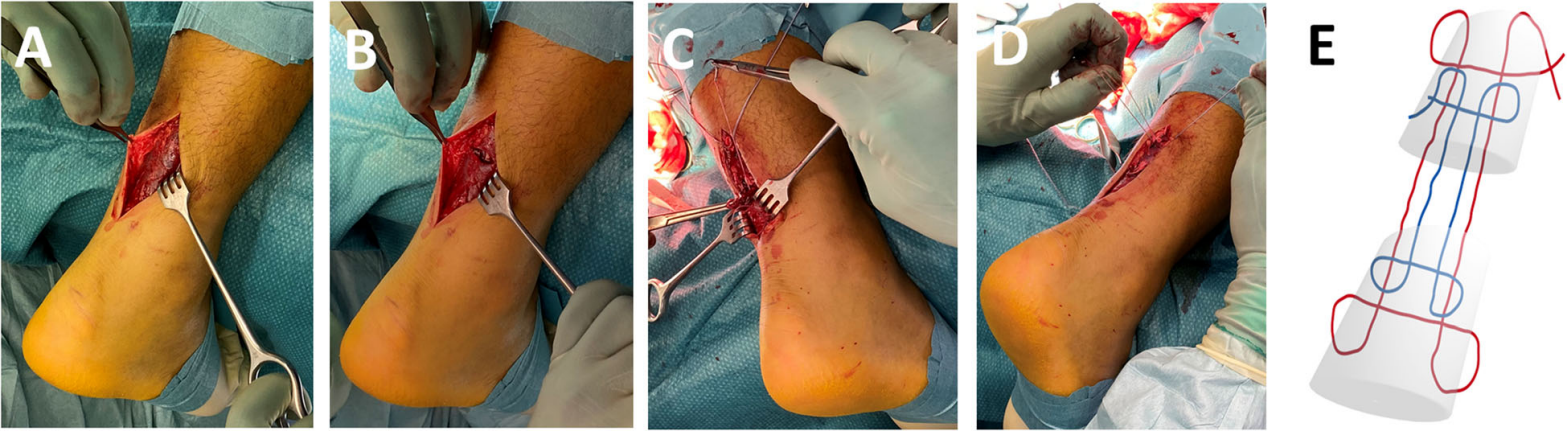
Illustration of the peritendium sparring surgical technique. **a** An incision of approximately 5 cm is placed over the rupture site. The peritendium is reached by careful stump dissection and is kept intact. **b** A transverse incision is made at the rupture site to expose the rupture. **c** The tendon stumps are drawn into the transverse incision and two modified Kessler sutures (Fiber-wire®, Arthrex size 2) are used to fix the tendon approximately 4 cm proximal and distal to the transverse incision. **d** The peritendium is closed with Vicryl, Ethicon 2–0 and the ankle is placed in maximal, unforced plantar flexion before the sutures are tightened maximally, bringing the tendon stumps together inside the peritendium. **e** A schematic drawing of the double Fiber-wire®, Arthrex size 2 a.m. Kessler with modification in terms of knots proximally on the sides

Participants destined for operative treatment, either through allocation to the operative group or as a result of allocation to the individualized treatment algorithm, are operated on within 14 days of the rupture. The procedure is performed under local anesthesia with the patient in the prone position. Preoperatively, 2 g of IV dicloxacillin are administered. An incision of approximately 5 cm is placed over the rupture site just medial to the midline. The peritendium is reached by stump dissection and is kept intact (Fig. 4). The rupture is palpated through the peritendium and a transverse incision is made to expose the rupture if needed. The tendon stumps are drawn into the transverse incision and two modified Kessler sutures (Fiber-wire®, Arthrex size 2) are used to fix the tendon approximately 4 cm proximal and distally to the transverse incision. The ankle is placed in maximal, unforced plantar flexion before the sutures are tightened maximally, bringing the tendon stumps together inside the peritendium. The peritendium is closed with Vicryl, Ethicon 2–0 (Fig. 4) [4]. After tensioning of the sutures, the ankle must be in an equinus position comparable with the uninjured leg or re-suturing needs to be performed. The skin is closed using single madras sutures. The lower leg is then placed in a circular below-the-knee cast with the ankle held at maximal comfortable plantar flexion. Weight bearing is not allowed and crutches are obligatory for the following 3 weeks. After 3 weeks from operative treatment the cast is removed in the outpatient clinic and the injured leg is transferred to a functional brace (Walker boot) with three heel wedges promoting 20° plantar flexion over the ankle (the same as for the non-operative treatment).

The participants will follow standard rehabilitation and the follow-up evaluations.

#### Rehabilitation

All participants in the trial are treated similarly in all aspects except for the initial operative or non-operative treatment.

#### Three weeks after surgery or 3 weeks after the non-operative treatment is started

Participants are seen in the outpatient clinic and the cast is removed. Sutures are removed for operatively treated patients. The leg is placed in the functional brace (Walker boot) with three heel wedges promoting 20° plantar flexion over the ankle. The participants are instructed to remove the consecutive two wedges after 2 and 4 weeks, respectively, and encouraged to start partial weight bearing of approximately 10–15 kg load from weeks 4 to 7 and full weight bearing from week 8. The brace must be kept on during sleep but can be removed during bathing if the leg can be kept completely off-loaded.

#### Nine weeks after surgery or 9 weeks after the non-operative treatment is started

At 9 weeks, the functional brace is removed and the tendon is examined by the physiotherapist. If the tendon has heeled as expected, exercises are started. The participants are recommended to use shoes with a heel wedge of minimum 10 mm until 4 months after the injury. Compression stockings of 15–20 mmHg (“Jobst active wear/ulcer”) are recommended for 6 months. At weeks 9–13 the participants are instructed to perform a written home exercise program two times every day. The program includes six exercises: the first two exercises have the purpose of increasing the range of motion in the ankle joint (pro- and supination and dorsal-plantar flexion 3 × 10 repetitions each), loaded motion over the ankle in plantar flexion with resistance from an elastic band (3 × 10 repetitions), side laying hip abduction (to activate m. gluteus medius, 2 × 15 repetitions), standing heel lift (3 × 10 repetitions), balance training (one leg stand 3 × 30 seconds). The participants are referred to continued rehabilitation in the municipality from week 13. To increase compliance the physiotherapists in the municipality are given written instructions on which exercises are recommended (progressive exercises for increased muscle strength, proprioception and balance in the ankle and leg, and training of walking ability). Prior to the start of the trial, all collaborating municipalities were invited to a meeting, where the rehabilitation process was described in depth by a physiotherapist and orthopedic surgeon at their hospital.

Instructions given to the participants at 9 weeks:

- Avoid total dorsiflexion with weight bearing. When dorsal flexing the ankle in standing, the vertical line from patella shall land behind the front line of the toes. Without weight bearing the patients are allowed to dorsiflex until the tendon is tightened.
- Use compression socks during daytime.
- If encountering problems with a swollen ankle or pain, take a break during the day, with the foot elevated.
- Rest if pain in the tendon.
- Walk with the crutches, as long as the patient is limping while walking.
- The participants are transferred to shoes for weight bearing for a minimum of 2 months with 1 cm heel rise. In running shoes with 1 cm heel lift an addition heel rise is not applicable.
- Cycling on a stationary bike can start immediately after the walker is removed. The pedal should be placed under the middle of the foot the first 2 months. Biking outside is allowed 12 weeks after injury.
- Swimming is allowed 11 weeks after injury (with great care getting into the water).
- Car driving is allowed 11 weeks after injury if the patient has injured the left leg and after 13 weeks if it is the right leg that is injured.

At 16 weeks, running on soft, even surfaces can be started slowly, if the participants can walk 5 m on tip-toe and perform five consecutive single heel rises on the injured leg (at 90% of the height of the uninjured leg). After 6–9 months, the participants can slowly start returning to contact sports but postpone participation in match and competition till after 12 months.

Concomitant care permitted or prohibited does not differ between the intervention groups.

### Follow- up evaluation

Follow-up is performed at 6 and 12 months at the outpatient clinic of the treating centers. Participants are contacted by telephone a couple of days prior to prevent drop out. At each evaluation appointment, the participants fill out the ATRS on a tablet.

All participants perform a warm up before testing: they ride a stationary bike for 5 min with minimal force and perform 3 × 10 single heel rises on both legs.

Firstly, the investigator will conduct ATRA followed by the heel-rise work test and registration of any complications or adverse effects. For this first part of the follow-up evaluation the participants will have tape placed on the back of the lower calf, so any surgical scare is covered in order to blind the investigator of the initial operative or non-operative treatment. Secondly, the investigator will conduct the CALM of both the total and the free part of the Achilles tendon, which is not possible to perform blinded.

### Registration and availability of data

All data are registered digitally in a web-based database (hosted by Procordo, Copenhagen, Denmark) especially designed for the trial. The registration forms are available from the first author upon request. The patients are given a patient identifier. All the results will be published at group level only and individual patients will not be able to be identified. At the completion of the study all identifiable data will be destroyed.

The project has been reported to the Danish Data Protection Agency, The Capital Region of Denmark, for all participating hospitals in the application (identifier AHH-2018-009, I-Suite no. 6356).

Data handling will be conducted in accordance with § 27 of the Danish Public Administration Act of patient confidentiality.

The patients will be informed, both verbally and in writing, that data are stored and analyzed in a computer, that the patient’s anonymity is preserved, and that the data protection legislation will be adhered to.

There will be free access to the final anonymized trial dataset. Due to the limited size of the study and the safety of the treatments, a data monitoring committee is not considered necessary.

#### Sample size

A difference in limb symmetry index for heel-rise work of 10% is considered clinically relevant. Based on the study by Silbernagel et al. [18], a standard deviation of 20 can be expected. The level of significance is set to *p* < 0.05 (C_2α_ = 1.96) and power is set at 90% (C_β_ = 1.281). The number of patients to be included in each treatment group is then calculated by: *N* = 2 × (C_2α_ + C_β_)^2^ × (SD)^2^/Δ^2^. Eighty-four patients have to be included in each group. Due to the risk of patient dropout, 100 will be included in each group. In total, 300 patients will be included. The incidence rate of patients with an acute Achilles tendon rupture at each hospital have been calculated and, based on previous experience, we expect being able to include 50% of patients passing through the departments.

#### Analysis of endpoints

Demographic parameters will be presented for each treatment group with mean and standard deviation (SD) or median and interquartile range (IQR) for continuous parameters and frequencies and percentages for categorical parameters.

Difference in Heel-rise work test will be tested by *t*-test or, if data can’t be assumed to be normally distributed, Wilcoxon sum rank test. Test will be made for the comparison individual vs operative treatment and individual vs non-operative treatment. To account for possible confounders, a linear regression will also be fitted for each comparison. Confounders include, but are not limited to, sex, age, BMI, and ATRS pre-injury; the confounding effect of these variables will be evaluated in the model by including and removing the variable and evaluating the change between the treatment group estimates.

Differences in severe complications (yes/no) between individual and operative treatment will be tested by chi-square test or, if the amount of expected observations for a single combination is less than 5, Fishers exact test. Additionally, the test will be adjusted for possible confounders in a logistic regression model; possible confounders and evaluation of these will be done as described in the Heel-rise work test models. Similar tests and models are made for the comparison of individual and non-operative treatments. Differences between individual and operative treatment and individual and non-operative treatment for all other secondary outcomes will be tested by *t*-test or Wilcoxon sum rank test.

All analysis will be done as intention-to-treat (ITT), that is, each patient is analyzed according to the treatment group they are randomized to. The results will therefore be interpreted as the effect of offering the patient the specific treatment options, regardless of if they complete them or not. Missing data will be imputed by multiple imputation, with imputation models based on available variables believed to be predictive of the missing measures. Additionally, the analysis for the primary outcome will be conducted as per protocol analysis, with per protocol defined as patients complying with the assigned protocol.

No interim analyses will be done.

### Safety

#### Risks and side effects

All participants in the study are at risk of developing pressure wounds from the functional brace and cast and a small risk of developing adhesion of the tendon to the skin. For participants receiving non-operative treatment there is a higher risk of re-rupture and an expected relatively higher risk of elongation of the injured Achilles tendon and inferior muscular force in the plantar flexion over the ankle joint compared to the patients receiving operative treatment. For the participants receiving operative treatment there is a risk of infection in the operative wound and temporary or permanent damage to the sural nerve.

The only possible need of modifying the allocated intervention for a given trial participant is if a participant treated non-operatively has insufficient healing after 12–16 weeks after injury or experiences a trauma re-injuring the tendon during the healing phase. In these situations, surgery is most often needed.

All participants are covered by the hospitals insurance during the treatment period.

#### Education and training

Observations and measurements are carried out by trained personnel at the orthopedic department or by trained sports physiotherapists at the participating centers. Follow-up evaluations are performed by the project physiotherapists, who are blinded to the initial treatment of operative or non-operative treatment (at all centers beside one where the physiotherapists see the patients all the time) until performing CALM, where the presence of a surgical scar is evident. The second author will also conduct test calibration once a year of the outcome measures within the doctors and physiotherapists that are involved in data collection.

#### Adverse events

In this context, adverse events are defined as any unintended, unfavorable finding, symptom, or disease that occurs, whether or not it is likely to be related to the study. All adverse events will be recorded, and special attention is paid to sural nerve damage, re-rupture rate, deep venous thrombosis, and both superficial and deep infections. Healing of the tendon in an elongated length is not considered an adverse event but is a primary endpoint of the study.

#### Critical adverse events

In this context, a critical adverse event is defined as an event or reaction which will cause death, life-threatening situations, hospitalization or prolongation of existing hospitalization, or permanent or severe disability, including an unacceptably high incidence of sural nerve damage.

An investigator assesses whether there is a reasonable possibility that the critical adverse event is possibly correlated with the trial’s modified treatment. The following factors are included in the assessment: consistency in time, consistency with the known effects of treatment, and alternative causes.

If a critical adverse event is considered to have a causal relationship with the treatment, then the project manager, those clinically responsible, and the other investigators will evaluate whether the study should be terminated.

### Satellite studies

Separate protocols have been written for the following sub-studies. The main study is referred to as study 1.

Study 2 *Achilles tendon elongation and gait pattern after rupture: A three-armed randomized controlled trial comparing an individualized treatment algorithm vs operative or non-operative treatment*. The first 60 participants included at Copenhagen University Hospital Amager-Hvidovre will have a closer and more intense follow-up. In addition to the tests performed in study 1, these participants will have MRI performed at 12 months and gait analysis performed at 6 and 12 months to determine if Achilles tendon elongation and gait pattern differ between participants treated using an individualized treatment algorithm and participants treated as usual (two control groups: participants treated operatively and non-operatively). The study is registered at ClinicalTrials.gov (NCT03543943).

Study 3 *Development of Achilles tendon elongation and its effect on physical function the first year after rupture: A prospective cohort study*. All participants from study 2 together with patients who do not wish to be included in the randomized controlled trial at Copenhagen University Hospital Amager-Hvidovre will be offered the opportunity to participate in a prospective cohort attending the same follow-up examinations as in study 2 but without MRI and gait analysis. The objective of this study is to examine how elongation of the Achilles tendon develops during the first year after rupture among patients with an Achilles tendon rupture, and how it affects physical function. The aim is to define a cutoff of acceptable elongation dividing the participants who obtain normal physical function (Limb Symmetry Index > 90%) from those who do not. The study is registered at ClinicalTrials.gov (NCT03525314).

## Discussion

The objective of this study is to investigate if an evidence-based individualized algorithm (CARTA) for treatment of acute Achilles tendon rupture gives a better functional outcome than treating all patients either non-operatively or operatively per default. The results will indicate if ultrasonography can help individualize the treatment for this group of patients.

CARTA present a way to structure the treatment decision for the patients with an Achilles tendon rupture. CARTA is easy to use in clinical practice and gives the clinician an objective tool when deciding if the patient should be recommended for operative or non-operative treatment.

The ultrasonographic measurements used in CARTA, both the extent of tendon overlap at the site of rupture as well as CALM, are easy to use and do not demand an experienced ultrasound examiner. The degree of tendon overlap has not been validated and can therefore not stand alone in an algorithm. On the contrary, CALM has been tested both for validity [19] and reliability [17]. The relative reliability is seen to be excellent, but CALM has a quite large measurement error on an individual level. When using CALM for evaluation of tendon elongation over time in our trial, the measurement error at the group level (0.6 cm) needs to be applied—changes below 0.6 cm might be an error within the measurement and not a real change in tendon elongation. The measurement error for CALM was taken into account when statistically calculating and defining the cut-off level of 7% used in the CARTA algorithm.

In both the original study describing CALM [19] and the reliability study [17], the position of the patient is described as laying prone on the examination table with 10–20° flexion of the knee and the same degrees of plantar flexion over the ankle [17]. When discussing the use of CALM during the follow-up appointment among the study group, it was decided to make a minor change to the position of the patient: having the feet hanging free (over the end) outside the edge of the table. This way, the tendon will be stretched and is thought to better reveal the actual length of the tendon as it is physiologically stretched in the resting position during the investigation. The argument for not using this position at all time points is that, during the initial examination, we aim to measure the displacement of tendon ends in the position it will heal in. The consequence of the different position of the ankles is not deemed to affect the reliability of the CALM.

The first study to propose the use of ultrasonography for treatment selection was Amlang et al. in 2011 [14]. Unfortunately, validity and reliability of the measure have not been published. Our experience with this ultrasonographic examination is that it was difficult to learn and to use in clinical practice, which made us insecure about our results. They describe the ultrasonographic classification in their article but no randomized treatment results for comparison [14]. In 2015, Hutchinson et al. presented an algorithm, also based on an ultrasonographic examination, and their treatment regime [15]. However, they did not have a control group and their follow-up was not optimal, having no valid functional outcome and a quite large loss to follow-up. This trial presents the only treatment algorithm including a valid and reliable measurement (CALM).

An Achilles tendon rupture is an injury that affects the physical function of the patient to a large extent. Therefore, the present trial has chosen a functional outcome as the primary outcome. In contrast, several previous randomized controlled trials have used patient-reported outcome measures as primary outcomes [4, 12, 31]. However, their results have shown the patients to have an acceptable score within the patient reported outcome measures but markedly low scores among the tests of physical function. An example is Olsson et al. [4], having a median ATRS value of 89 points (100 being top score) but the heel-rise work test showed the injured leg performing at 45% compared to the non-injured leg. Having a functional outcome as primary outcome might, therefore, detect the functional deficits post-injury that the patient-reported outcome measures are not always able to capture.

The power of 0.9 and having two control groups is a strength within the design. To complete the trial within a reasonable time span and to improve the external validity of the trial, a multi-center setup was chosen. The improved external validity makes the results easier to implement and generalize.

When designing this trial, we prioritized a set up that would be feasible to implement into clinical practice. Therefore, the patients are following the initial standardized treatment plans at the hospital and then continue the rehabilitation and exercise program together with the physiotherapists in the municipality from week 13. Each of the hospitals in the study collaborates with up to 11 different municipalities and some patients also decide to visit a private physiotherapist. Before starting the project, all collaborating municipalities were invited to a meeting where information was given about the study and where a description of the rehabilitation program was provided as well as suggested exercises with progression were proposed. So even though we could not control the rehabilitation from week 13, we did try to advise the collaborating physiotherapists.

### Trial status

This is a report of the second version of the trial protocol dated 12 December 2018. Recruitment of patients began in June 2018 and is ongoing. It was initially estimated to take 2 years but due to logistical challenges the inclusion period is now expected to span 3–4 years (approximately February 2022). As of 21st February 2020, 108 out of 300 patients had been included.

## Supplementary information

**Additional file 1.** SPIRIT 2013 Checklist for the ReTrain pilot RCT: Recommended items to address in a clinical trial protocol and related documents.

## Data Availability

The final anonymized dataset will be available from the author MTV upon request.

## Abbreviations

CARTA: Copenhagen Achilles Rupture Treatment Algorithm
CALM: Copenhagen Achilles Length Measure
ATRS: Achilles tendon Total Rupture Score
ATRA: Achilles Tendon Resting Angle
MRI: Magnetic resonance imaging
ASA: American Society of Anesthesiologists
US: Ultrasonographic
BMI: Body mass index
ITT: Intention to treat

## References

[1] Ganestam A, Kallemose T, Troelsen A, Barfod KW (2016). Increasing incidence of acute Achilles tendon rupture and a noticeable decline in surgical treatment from 1994 to 2013. A nationwide registry study of 33,160 patients. Knee Surg Sports Traumatol Arthrosc.

[2] Willits K, Amendola A, Bryant D, Mohtadi NG, Giffin JR, Fowler P (2010). Operative versus nonoperative treatment of acute Achilles tendon ruptures: a multicenter randomized trial using accelerated functional rehabilitation. J Bone Joint Surg Am.

[3] Erickson BJ, Mascarenhas R, Saltzman BM, Walton D, Lee S, Cole BJ (2015). Is operative treatment of Achilles tendon ruptures superior to nonoperative treatment? A systematic review of overlapping meta-analyses. Orthop J Sport Med.

[4] Olsson N, Silbernagel KG, Eriksson BI, Sansone M, Brorsson A, Nilsson-Helander K (2013). Stable surgical repair with accelerated rehabilitation versus nonsurgical treatment for acute achilles tendon ruptures: A randomized controlled study. Am J Sports Med.

[5] Mundi R, Madden K, Bhandari M (2014). Cochrane in CORR®: Surgical interventions for treating acute Achilles tendon ruptures (review). Clin Orthop Relat Res.

[6] Khan RJ, Carey Smith RL (2010). Surgical interventions for treating acute Achilles tendon ruptures. Cochrane Database Syst Rev.

[7] Barfod KW, Bencke J, Lauridsen HB, Dippmann C, Ebskov L, Troelsen A (2015). Nonoperative, dynamic treatment of acute achilles tendon rupture: Influence of early weightbearing on biomechanical properties of the plantar flexor muscle-tendon complex-a blinded, randomized, controlled trial. J Foot Ankle Surg.

[8] Mark-Christensen T, Troelsen A, Kallemose T, Barfod KW (2016). Functional rehabilitation of patients with acute Achilles tendon rupture: a meta-analysis of current evidence. Knee Surg Sports Traumatol Arthrosc.

[9] Barfod KW, Bencke J, Lauridsen HB, Ban I, Ebskov L, Troelsen A (2014). Nonoperative dynamic treatment of acute achilles tendon rupture: the influence of early weight-bearing on clinical outcome: a blinded, randomized controlled trial. J Bone Joint Surg Am.

[10] Barfod KW, Nielsen F, Helander KN, Mattila VM, Tingby O, Boesen A (2013). Treatment of acute achilles tendon rupture in scandinavia does not adhere to evidence-based guidelines: A cross-sectional questionnaire-based study of 138 departments. J Foot Ankle Surg.

[11] Silbernagel KG, Steele R, Manal K (2012). Deficits in heel-rise height and Achilles tendon elongation occur in patients recovering from an Achilles tendon rupture. Am J Sports Med.

[12] Nilsson-Helander K, Silbernagel KG, Thomeé R, Faxén E, Olsson N, Eriksson BI (2010). Acute achilles tendon rupture: a randomized, controlled study comparing surgical and nonsurgical treatments using validated outcome measures. Am J Sports Med.

[13] Bergkvist D, Åström I, Josefsson PO, Dahlberg LE (2012). Acute achilles tendon rupture: A questionnaire follow-up of 487 patients. J Bone Jt Surg.

[14] Amlang MH, Zwipp H, Friedrich A, Peaden A, Bunk A, Rammelt S (2011). Ultrasonographic classification of achilles tendon ruptures as a rationale for individual treatment selection. ISRN Orthop.

[15] Hutchison AM, Topliss C, Beard D, Evans RM, Williams P (2015). The treatment of a rupture of the Achilles tendon using a dedicated management programme. Bone Joint J.

[16] Barfod KW, Riecke AF, Hansen P, Maier JF, Boesen A, Døssing S (2015). Validation of a novel ultrasound measurement of achilles tendon length. Knee Surg Sport Traumatol Arthrosc.

[17] Hansen MS, Kristensen MT, Budolfsen T, Ellegaard K, Hölmich P, Barfod KW (2020). Reliability of the Copenhagen Achilles length measure (CALM) on patients with an Achilles tendon rupture. Knee Surg Sport Traumatol Arthrosc.

[18] Silbernagel KG, Nilsson-Helander K, Thomeé R, Eriksson BI, Karlsson J (2010). A new measurement of heel-rise endurance with the ability to detect functional deficits in patients with Achilles tendon rupture. Knee Surg Sports Traumatol Arthrosc.

[19] Barfod KW, Riecke AF, Boesen A, Hansen P, Maier JF, Døssing S (2014). Validation of a novel ultrasound measurement of achilles tendon length. Knee Surg Sport Traumatol Arthrosc.

[20] Brouwer EF, Myhrvold SB, Benth JŠ, Hoelsbrekken SE (2018). Ultrasound measurements of Achilles tendon length using skin markings are more reliable than extended-field-of-view imaging. Knee Surg Sport Traumatol Arthrosc.

[21] Barfod KW, Riecke AF, Boesen A, Hansen P, Maier JF, Doessing S (2018). Validity and reliability of an ultrasound measurement of the free length of the Achilles tendon. Dan Med J.

[22] Carmont MR, Grävare Silbernagel K, Brorsson A, Olsson N, Maffulli N, Karlsson J (2015). The Achilles tendon resting angle as an indirect measure of Achilles tendon length following rupture, repair, and rehabilitation. Asia-Pacific J Sport Med Arthrosc Rehabil Technol.

[23] Hansen MS, Barfod KW, Kristensen MT. Development and reliability of the Achilles Tendon Length Measure and comparison with the Achilles Tendon Resting Angle on patients with an Achilles tendon rupture. Foot Ankle Surg. 2017;23(4):275–80. 10.1016/j.fas.2016.08.002. 10.1016/j.fas.2016.08.002 29202987

[24] Nilsson-Helander K, Thomeé R, Silbernagel KG, Thomeé P, Faxén E, Eriksson BI (2007). The Achilles tendon Total Rupture Score (ATRS): development and validation. Am J Sports Med.

[25] Ganestam A, Barfod K, Klit J, Troelsen A (2013). Validity and reliability of the Achilles Tendon Total Rupture Score. J Foot Ankle Surg.

[26] Tegner Y, Lysholm J (1985). Rating systems in the evaluation of knee ligament injuries. Clin Orthop Relat Res.

[27] Speedtsberg MB, Kastoft R, Barfod KW, Penny JØ, Bencke J (2019). Gait function and postural control 4.5 years after nonoperative dynamic treatment of acute Achilles tendon ruptures. Orthop J Sport Med.

[28] Simmonds FA (1957). The diagnosis of the ruptured Achilles tendon. Practitioner.

[29] Matles AL (1975). Rupture of the tendo achilles: another diagnostic sign. Bull Hosp Joint Dis.

[30] Arverud ED, Anundsson P, Hardell E, Barreng G, Edman G, Latifi A (2016). Ageing, deep vein thrombosis and male gender predict poor outcome after acute Achilles tendon rupture. Bone Joint J.

[31] Barfod KW, Hansen MS, Hölmich P, Kristensen MT, Troelsen A. Efficacy of early controlled motion of the ankle compared with immobilisation in nonoperative treatment of patients with an acute Achilles tendon rupture: an assessor-blinded, randomised controlled trial. Br J Sports Med. 2019. 10.1136/bjsports-2019-100709. [Epub ahead of print]. 10.1136/bjsports-2019-100709 31597624

